# Prevalence and stabilizing trends in overweight and obesity among children and adolescents in China, 2011-2015

**DOI:** 10.1186/s12889-018-5483-9

**Published:** 2018-05-02

**Authors:** Jiguo Zhang, Huijun Wang, Zhihong Wang, Wenwen Du, Chang Su, Ji Zhang, Hongru Jiang, Xiaofang Jia, Feifei Huang, Yifei Ouyang, Yun Wang, Bing Zhang

**Affiliations:** National Institute for Nutrition and Health, Chinese Center for Disease Control and Prevention, No.29 Nanwei Road, Beijing, 100050 China

**Keywords:** Adolescents, Children, China, Obesity, Overweight

## Abstract

**Background:**

The prevalence of childhood overweight and obesity in developed countries appears to be plateauing. The purpose of this study was to provide the most recent data on the prevalence and trends in overweight and obesity among Chinese children and adolescents from 2011 to 2015.

**Methods:**

We used data collected in the China Health and Nutrition Survey (CHNS) and China Nutritional Transition Cohort Study (CNTCS). We used two waves of the survey in 12 provinces conducted in 2011 (aged 7–18 years; *n* = 1458) and 2015 (aged 7–18 years; *n* = 1084) to perform a trend analysis. We used data collected in 15 provinces (aged 7–18 years; *n* = 1617) to estimate the prevalence of overweight and obesity among Chinese children and adolescents in 2015.

**Results:**

In 2015, based on the Working Group for Obesity in China (WGOC) criteria, the prevalence of overweight and obesity were 14.0% (95% CI, 11.6–16.3) and 10.5% (95% CI, 8.4–12.6) in boys, and 9.7% (95% CI, 7.7–11.8) and 7.1% (95% CI, 5.2–8.9) in girls, respectively. The increase in BMI z-scores from 2011 to 2015 was statistically significant among adolescents (*p* = 0.0083), but not among children. No significant changes were observed in prevalence of overweight and obesity between 2011 and 2015, excepting adolescents aged 12–18 years (*p* = 0.0086).

**Conclusions:**

Since 2011, overweight has remained stable, and obesity has stabilized in children, though not in adolescents. Although levels of childhood overweight and obesity in China are not high compared to other developed countries, they remain concerning enough that effective policies and interventions need to be sustained and intensified for lowering rates of childhood overweight and obesity.

## Background

Childhood obesity is a serious public health problem that requires urgent attention [1]. Today many children are growing up in an obesogenic environment that encourages weight gain and obesity [2]. It is necessary and important to build monitoring systems to regularly track the prevalence of childhood obesity, thus providing data for policy development and in offering evidence of the impact and effectiveness of interventions [1].

Prevalence of obesity has increased substantially in children and adolescents around the world, including in China [3, 4]. The childhood overweight and obesity epidemic in China began in 1985, and at that time, the prevalence of overweight (1.1%) and obesity (0.1%) was very low [5]. Afterwards, rapid economic development in China caused transitions in nutrition and physical activity behavior, which contributed to a rapid increase in the prevalence of overweight and obesity [6–8]. In 2012, the national overweight and obesity prevalence of children aged 6–17 years were 9.6% and 6.4%, respectively [9]. However, recent evidence suggests that a plateaued trend or even a decrease in childhood overweight and obesity is underway in many developed countries. For example, in Germany, researchers observed a further stabilization of overweight and obesity prevalence rates for all age groups and a decrease in rates for younger ages (4–7·99 years, 8–11·99 years) [10]. Similarly, stabilized trends have been observed in other countries, such as US children aged 6 to 11 years [11], UK children aged 2 to 10 years [12], Australian primary school children [13], and Canadian children [14].

Many trend analyses have shown a continuously rising prevalence of obesity/overweight in China, with more rapid increases in toddlers and boys than in their counterparts, as well as in rural versus urban areas [4, 15–20]. However, most previous studies were based either on earlier surveys or on provincial data. In this study, our objectives were to provide the most recent estimates of obesity and overweight prevalence among Chinese children and adolescents in 2015, to evaluate trends in BMI z-scores and to examine the trends in prevalence of overweight and obesity for the 5 years of two adjacent surveys in 2011 and 2015. Additionally, we present more detailed trend analyses by age, sex, living area and geographic region.

## Methods

### Study design and subjects

We used data collected in the China Health and Nutrition Survey (CHNS) and China Nutritional Transition Cohort Study (CNTCS). CHNS was an ongoing large-scale, longitudinal, household-based survey of 10 waves (1989–2015) [21, 22]. Beginning in 2011, the CHNS covered 12 provinces that varied in demography, geography, economic development, and public resources. In 2015, the CNTCS began in the additional provinces of Zhejiang, Shanxi, and Yunnan. In each province, a multistage, stratified, random cluster sampling design was used to ensure a suitable representation of the population. Two cities and four counties were selected by income (low, middle, and high). Four communities from each city/county were selected randomly; in each community, 20 households were randomly selected and followed in ten subsequent waves. In some cases, new households were recruited to substitute for those who migrated out of the community for different reasons.

The trend analysis was based on two waves of the survey in 12 provinces conducted in 2011 and 2015. Our sample included children and adolescents aged 7 to 18 years from each family included in the two rounds of survey. There were 1458 and 1084 children and adolescents with complete demographic and physical examinations, respectively. We used data combining CHNS with CNTCS collected in 15 provinces to estimate the prevalence of overweight and obesity among children and adolescents in 2015.

### Measures and definition

Anthropometrical measurements were conducted by well-trained health workers who followed a reference protocol recommended by the World Health Organization (WHO) [23]. Height was measured to the nearest 0.1 cm without shoes using a portable SECA206 wall-mounted metal tape, and weight was measured to the nearest 0.1 kg with lightweight clothing on a beam SECA 880 in 2011 and a TANITA BC601 in 2015. Both the scales and stadiometers were calibrated before use. Body mass index (BMI) was calculated as weight in kilograms divided by height in meters squared.

The sex- and age-specific BMI cutoff points recommended by the International Obesity Task Force (IOTF) [24], WHO [25], and Working Group for Obesity in China (WGOC) were used to define overweight and obesity. The former two sets of criteria have been used internationally to assess and compare childhood overweight and obesity in different countries. Considering the differences in body composition across different ethnic groups, the WGOC, which was organized by the International Life Science Institute Focal Point in China, conducted an analysis of BMI of children and adolescents aged 7–18 years. Overweight was defined as BMI >85th percentile but ≤95th percentile, relative to gender and age, whereas obesity was defined as BMI >95th percentile, and a new BMI classification reference was recommended by the WGOC in 2004 [26]. This standard is the most appropriate one that showed its superiority in both prospectively and actuality, and was consistent with the Eastern Asia ethnic characteristics of body fatness growth [27]. BMI-for-age z-score is a quantitative measure of the deviation of a specific BMI value from the mean of that population, and was calculated with the WHO (WHO 2007) references.

### Ethical approval

All subjects’ parents gave written informed consent for their children’s participation in the survey. The CHNS was approved by the Institutional Review committees of the University of North Carolina at Chapel Hill and the National Institute for Nutrition and Health, Chinese Center for Disease Control and Prevention (No. 201524).

### Statistical analysis

The primary data analysis was conducted in 2017. We presented prevalence estimates for overweight and obesity in different survey years according to age, gender, living area and geographic region groups. Living area of urban and rural was based on the Chinese administrative division. A line running along the Qinling Mountains and Huaihe River was considered as the natural boundary between north and south of geographic region in China.

T tests were applied to test differences between groups and trends for BMI z-scores. Chi-square analyses were conducted to assess differences in prevalence of overweight and obesity for categorical variables. All statistical analyses were performed using version 9.2 of the SAS software package (SAS Institute, Inc. Cary, NC, USA). Statistical significance was defined as *P* < 0.05.

## Results

### Prevalence of overweight and obesity

Table 1 shows the prevalence of overweight and obesity among Chinese children and adolescents aged 7–18 years in 2015. Using IOTF, WHO, and WGOC criteria, the prevalence of overweight and obesity were 13.1% (95% CI, 11.5–14.8) and 5.7% (95% CI, 4.5–6.8), 15.5% (95% CI, 13.7–17.2) and 8.8% (95% CI, 7.4–10.2), 11.9% (95% CI, 10.4–13.5) and 8.8% (95% CI, 7.5–10.2), respectively. We found a much higher prevalence of obesity among boys than among girls (*p* < 0.0001, p < 0.0001, *p* = 0.0143). The northern children and adolescents showed higher prevalence of overweight and obesity than their southern peers, except for overweight using the WGOC criteria. In addition, the IOTF criteria found a higher prevalence of obesity among urban children and adolescents (*p* = 0.0458), the WGOC criteria found a much higher prevalence of overweight among boys than among girls (*p* = 0.0087). There was no significant difference between age groups by the three sets of criteria.

**Table 1:** Prevalence of overweight and obesity among Chinese children and adolescents aged 7-18y in 2015 by IOTF, WHO and WGOC criteria

		IOTF criteria, % (95% CI)				WHO criteria, % (95% CI)				WGOC criteria, % (95% CI)			
	*N*	Overweight	*p*	Obesity	*p*	Overweight	*p*	Obesity	*p*	Overweight	*p*	Obesity	*p*
All	1617	13.1 (11.5–14.8)		5.7 (4.5–6.8)		15.5 (13.7–17.2)		8.8 (7.4–10.2)		11.9 (10.4–13.5)		8.8 (7.5–10.2)	
Age													
7–11	1007	13.2 (11.1–15.3)	0.8821	5.7 (4.2–7.1)	0.9481	16.0 (13.7–18.2)	0.4511	9.6 (7.8–11.4)	0.1204	11.7 (9.7–13.7)	0.7286	9.0 (7.3–10.8)	0.7252
12–18	610	13.0 (10.3–15.6)		5.7 (3.9–7.6)		14.6 (11.8–17.4)		7.4 (5.3–9.5)		12.3 (9.7–14.9)		8.5 (6.3–10.7)	
Gender													
Boys	837	14.0 (11.6–16.3)	0.2842	7.9 (6.1–9.7)	< 0.0001	17.1 (14.5–19.6)	0.0613	12.9 (10.6–15.2)	< 0.0001	14.0 (11.6–16.3)	0.0087	10.5 (8.4–12.6)	0.0143
Girls	780	12.2 (9.9–14.5)		3.3 (2.1–4.6)		13.7 (11.3–16.1)		4.4 (2.9–5.8)		9.7 (7.7–11.8)		7.1 (5.2–8.9)	
Area													
Urban	435	15.2 (11.8–18.5)	0.1362	7.6 (5.1–10.1)	0.0458	18.2 (14.5–21.8)	0.0685	10.6 (7.7–13.5)	0.1222	14.3 (11.0–17.5)	0.0812	10.8 (7.9–13.7)	0.0920
Rural	1182	12.4 (10.5–14.2)		5.0 (3.7–6.2)		14.5 (12.5–16.5)		8.1 (6.6–9.7)		11.1 (9.3–12.9)		8.1 (6.6–9.7)	
Region													
North	499	16.8 (13.5–20.1)	0.0030	9.0 (6.5–11.5)	0.0001	19.2 (15.8–22.7)	0.0050	13.6 (10.6–16.6)	< 0.0001	14.2 (11.2–17.3)	0.0575	13.6 (10.6–16.6)	< 0.0001
South	1118	11.4 (9.6–13.3)		4.2 (3.0–5.4)		13.8 (11.8–15.8)		6.6 (5.2–8.1)		10.9 (9.1–12.7)		6.7 (5.2–8.2)	

*IOTF* International Obesity Task Force, *WHO* World Health Organization, *WGOC* Working Group for Obesity in China

### Trends in BMI z-scores among children and adolescents

Table 2 shows the mean BMI z-scores among Chinese children and adolescents aged 7–18 years between 2011 and 2015. The increase in BMI z-score from 2011 to 2015 was statistically significant (*p* = 0.0211). After stratifying by age group, gender, living area and region, we found a significant increase in BMI z-scores among adolescents aged 12–18 (*p* = 0.0083), boys (*p* = 0.0107), urban (*p* = 0.0173) and southern children and adolescents (p = 0.0087).

**Table 2:** Trends in BMI z-scores among Chinese children and adolescents aged 7-18y: 2011–2015

		2011		2015	*P*
	*N*	Mean ± SD	*N*	Mean ± SD	
All	1458	−0.0749 ± 1.3632	1084	0.0528 ± 1.4028	0.0211
Age					
7–11	762	0.0421 ± 1.5078	698	0.0737 ± 1.4251	0.6818
12–18	696	−0.2031 ± 1.1726	386	0.0150 ± 1.3626	0.0083
Gender					
Boys	743	0.0426 ± 1.4529	554	0.2522 ± 1.4710	0.0107
Girls	715	−0.1970 ± 1.2527	530	−0.1556 ± 1.2968	0.5700
Area					
Urban	440	0.1275 ± 1.3571	282	0.3732 ± 1.3381	0.0173
Rural	1018	−0.1625 ± 1.3572	802	−0.0599 ± 1.4085	0.1155
Region					
North	461	0.3738 ± 1.3626	302	0.4911 ± 1.4773	0.2612
South	997	−0.2824 ± 1.3133	782	−0.1165 ± 1.3360	0.0087

### Trends in prevalence of overweight and obesity

Table 3 shows the trends in prevalence of overweight and obesity among Chinese children and adolescents aged 7–18 years using the WGOC criteria between 2011 and 2015. We found no significant difference in prevalence of overweight and obesity between 2011 and 2015, except for adolescents aged 12–18 years, who had a higher prevalence of obesity in 2015 than that in 2011 (*p* = 0.0086).

**Table 3:** Trends in Prevalence of overweight and obesity among Chinese children and adolescents aged 7-18y by WGOC criteria: 2011–2015

	2011	2015		Overweight		*p*	Obesity		*p*
	*N*	*N*		2011	2015		2011	2015	
All	1458	1084		10.6	11.8	0.3507	8.4	9.6	0.3113
Age									
7–11	762	698		10.6	11.0	0.8051	11.4	9.7	0.2993
12–18	696	386		10.6	13.2	0.2034	5.2	9.3	0.0086
Gender									
Boys	743	554		13.6	13.7	0.9483	9.2	11.7	0.1296
Girls	715	530		7.6	9.8	0.1579	7.7	7.4	0.8255
Area									
Urban	440	282		14.1	13.8	0.9214	9.8	13.1	0.1621
Rural	1018	802		9.1	11.1	0.1661	7.9	8.4	0.7001
Region									
North	461	302		15.0	15.9	0.7283	13.9	16.6	0.3111
South	997	782		8.6	10.2	0.2482	5.9	6.9	0.3966

*WGOC* Working Group for Obesity in China

## Discussion

Our findings show that the prevalence of overweight and obesity have remained stable among Chinese children since 2011, but not among adolescents aged 12–18 years for whom the prevalence of obesity has significantly increased between 2011 and 2015, consistent with findings from a recent Australian study [13]. The increase and higher prevalence of obesity in adolescents is concerning given the high likelihood of BMI tracking from adolescence into adulthood [28]. Thus, adolescents should be the key target of obesity prevention.

According to two reviews, the prevalence of childhood overweight and obesity appears to have stabilized since the late 1990s in many developed countries [29, 30]. In Asia, there was a decrease in the prevalence of overweight/obesity among Japanese schoolchildren from 2003 to 2012 [31]. In Korea, the prevalence of childhood overweight and obesity generally stabilized from 2001 to 2012 in both boys and girls [32]. In general, this stabilization in the trend has been attributed to the growing population concern about childhood obesity and continuous efforts of the family, school, community and government toward the prevention and treatment of childhood obesity [33]. As a result of these efforts, a whole series of interventions has been implemented to halt the alarming rise in the prevalence of childhood obesity, such as reducing television viewing duration and consumption of energy-dense foods, as well as increasing physical activity [33, 34].

Similarly, the Chinese government began to realize the importance of obesity control about 15 years ago, and implemented laws and regulations aimed at obesity prevention [35]. For example, China has already developed some guidelines to control general childhood obesity, such as Guidelines on Snacks for Chinese Children and Adolescents (published in 2008) and the School-age Children and Teenagers Overweight and Obesity Prevention and Control Guidelines (issued in 2007) [35]. Also, a large number of intervention programs have been initiated or were ongoing in the past 15 years, such as ‘Happy 10 minutes’. The key measures of program and policy options for prevention that began during childhood may have contributed to the stabilization in prevalence of childhood overweight and obesity. It is also possible that this flattening represents a temporary lull and that without continued efforts we may again see a rise in prevalence. Further studies are needed to assess the possible reasons of the trends in overweight and obesity.

In this study, we used three different sets of criteria to estimate the prevalence of overweight and obesity among Chinese children and adolescents in 2015. Among the three sets of criteria, the WHO criteria yielded the highest prevalence of overweight and obesity, while the WGOC criteria produced the lowest overweight prevalence, and the lowest obesity prevalence was generated by the IOTF criteria. These results from the three sets of criteria were expected, considering their use of different BMI cut-points for overweight and obesity among children and adolescents. Because different criteria are used throughout the world, it can be difficult to compare rates in China with those in other countries. Nonetheless, estimates of overweight and obesity among Chinese children and adolescents tend to be lower than in developed countries [3, 11]. However, childhood overweight and obesity is becoming a topic of great concern in China due to the higher prevalence in some provinces [36, 37] approaching that of Western countries.

In line with previous studies in China [6, 20], we found a gender difference in that boys showed a higher prevalence of obesity than girls. Differences in genetic and environmental factors could partly contribute to the gender differences in overweight and obesity. Moreover, there exists a traditional preference for boys in Chinese society, especially in rural areas [20]. Owing to this preference, boys are likely to enjoy more of the family’s resources and have less work than girls. In addition, obesity is often viewed as a characteristic of good health in boys [18]. On the other hand, girls are expected to maintain a slender shape, and as a result they pay more attention to weight control [6, 18].

In the present study, no significant urban-rural disparity was observed except the obesity seen using IOTF criteria, which was inconsistent with previous results showing that urban childhood had a higher prevalence of overweight and obesity than rural counterparts [6, 18]. In the past 15 years, the pace of the obesity epidemic in rural areas has been much faster than in urban areas, and thus the urban–rural disparity of obesity prevalence has been getting narrower, which means the burden of overweight and obesity may shift from the rich to the poor [16, 38]. These findings could be due to economic growth and changes in lifestyle. Family income and urbanization may be linked to dietary factors, and to factors associated with sedentary behavior such as increased television watching [39]. Research indicates that as income has improved, particularly in the low- and middle-income groups, the structure of the Chinese diet has shifted away from high-carbohydrate foods toward high-fat, high-energy-density foods [40]. Therefore, policymakers and experts should develop specific interventions for obesity in rural areas, even though their obesity prevalence is still lower than that in urban areas.

In the present study, we also found a north-south disparity in the prevalence of overweight and obesity. Northern children showed a higher prevalence of overweight and obesity than their southern counterparts. Climate could serve as one explanation for this disparity. The northern region is characterized by a longer winter with the coldest temperature is approximately below 0 °C. In southern cities, the winter is shorter with the coldest temperature above 0 °C. As a result, individuals in northern areas might exhibit decreased physical activity, and may store more fat to resist the cold temperatures in the winter [20]. Diet is another possible explanation. A previous study reported that childhood obesity was positively associated with traditional northern dietary pattern in China, which could be partly explained by the high carbohydrate intake and lower levels of micronutrients [41].

One important limitation of our study was the lack of large sample size to obtain nationally representative results, even though the survey captured different demographic and geographical areas. Second, because this study used data from two cross-sectional surveys, the data can’t be used to infer causality or predict future trends. Prospective studies could confirm or broaden our results. Finally, we did not analyze factors such as dietary and lifestyle habits that may affect trends in overweight and obesity.

Despite these limitations, to the best of our knowledge, this is the first study to report the most recent prevalence and stabilizing trends in overweight and obesity among Chinese children and adolescents using large survey data between 2011 and 2015. Based on the results, the government and scientific community could take appropriate measures for childhood obesity prevention. Besides, we used three different set of criteria to estimate the prevalence of childhood overweight and obesity, making international comparisons possible.

## Conclusion

In summary, overweight has remained stable since 2011, and obesity has stabilized in children, but not in adolescents. Continuing research is needed to confirm these trends. Effective policies and interventions need to be sustained and intensified to lower rates of childhood overweight and obesity, even though the prevalence remains lower than developed countries.

## Abbreviations

CHNS: China Health and Nutrition Survey
CNTCS: China Nutritional Transition Cohort Study
IOTF: International Obesity Task Force
WGOC: Working Group for Obesity in China
WHO: World Health Organization
